# Combination of ^18^F-FDG PET/CT and convex probe endobronchial ultrasound elastography for intrathoracic malignant and benign lymph nodes prediction

**DOI:** 10.3389/fonc.2022.908265

**Published:** 2022-08-05

**Authors:** Xinxin Zhi, Xiaoyan Sun, Junxiang Chen, Lei Wang, Lin Ye, Ying Li, Wenhui Xie, Jiayuan Sun

**Affiliations:** ^1^ Department of Respiratory Endoscopy, Shanghai Jiao Tong University, Shanghai, China; ^2^ Department of Respiratory and Critical Care Medicine, Shanghai Chest Hospital, Shanghai Jiao Tong University, Shanghai, China; ^3^ Shanghai Engineering Research Center of Respiratory Endoscopy, Shanghai, China; ^4^ Department of Nuclear Medicine, The Fifth People’s Hospital of Shanghai Fu Dan University, Shanghai, China; ^5^ Department of Nuclear Medicine, Shanghai Chest Hospital, Shanghai Jiao Tong University, Shanghai, China; ^6^ Department of Ultrasound, Shanghai Chest Hospital, Shanghai Jiao Tong University, Shanghai, China

**Keywords:** PET/CT, endobronchial ultrasound, elastography, lymph nodes, diagnosis

## Abstract

**Background:**

Positron emission tomography–computed tomography (PET/CT) and convex probe endobronchial ultrasound (CP-EBUS) elastography are important diagnostic methods in predicting intrathoracic lymph nodes (LNs) metastasis, but a joint analysis of the two examinations is still lacking. This study aimed to compare the diagnostic efficiency of the two methods and explore whether the combination can improve the diagnostic efficiency in differentiating intrathoracic benign LNs from malignant LNs.

**Materials and Methods:**

LNs examined by EBUS-guided transbronchial needle aspiration (EBUS-TBNA) and PET/CT from March 2018 to June 2019 in Shanghai Chest Hospital were retrospectively analyzed as the model group. Four PET/CT parameters, namely, maximal standardized uptake value mean standardized uptake value (SUVmean), SUVmean, metabolic tumor volume (MTV), and tumor lesion glycolysis (TLG); four quantitative elastography indicators (stiff area ratio, mean hue value, RGB, and mean gray value); and the elastography grading score of targeted LNs were analyzed. A prediction model was constructed subsequently and the dataset from July to November 2019 was used to validate the diagnostic capability of the model.

**Results:**

A total of 154 LNs from 135 patients and 53 LNs from 47 patients were enrolled in the model and validation groups, respectively. Mean hue value and grading score were independent malignancy predictors of elastography, as well as SUVmax and TLG of PET/CT. In model and validation groups, the combination of PET/CT and elastography demonstrated sensitivity, specificity, positive and negative predictive values, and accuracy for malignant LNs diagnosis of 85.87%, 88.71%, 91.86%, 80.88%, and 87.01%, and 94.44%, 76.47%, 89.47%, 86.67%, and 88.68%, respectively. Moreover, elastography had better diagnostic accuracies than PET/CT in both model and validation groups (85.71% *vs*. 79.22%, 86.79% *vs*. 75.47%).

**Conclusion:**

EBUS elastography demonstrated better efficiency than PET/CT and the combination of the two methods had the best diagnostic efficacy in differentiating intrathoracic benign from malignant LNs, which may be helpful for clinical application.

## Introduction

Positron emission tomography–computed tomography (PET/CT) plays a great role in the staging of mediastinal lymph nodes (LNs) of non-small cell lung cancer, with a sensitivity and specificity of 77% and 86%, respectively, for predicting LNs metastasis (1–3). However, PET/CT can only reflect the uptake value of contrast agent. Inflammatory changes in lymphoid follicles and histiocytes can increase ^18^F-fluorodeoxyglucose (^18^F-FDG) uptake, leading to the existence of false-positive results (4, 5). LNs with tuberculosis infection can also have a false-positive result because glucose metabolism increases with the accumulation of FDG in inflammatory phagocytes of granulomatous tissue (6). With the development of PET/CT technology and the increasing clinical demand for sensitivity of malignant LN prediction, the diagnostic false-positive rate (FPR) increased, leading to a very important pathological biopsy (7).

Endobronchial ultrasound-guided transbronchial needle aspiration (EBUS-TBNA) is an essential minimally invasive examination, and it can be used to diagnose mediastinal enlarged LNs with significantly high FDG uptake caused by anthracosis (8). For malignant diseases, due to the limited sampling of puncture needle, tumor micrometastasis may lead to the presence of false-negative results (9). Therefore, EBUS-TBNA has a false-negative rate (FNR) of 20% for lung cancer (10). Relevant guidelines indicate that sonographic features can be used to predict malignant and benign LNs during EBUS-TBNA operation, and may prevent the need for repeat EBUS procedures when initial biopsy results are inconclusive (11, 12). Elastography can quantify the degree of tissue deformation in grayscale mode and relative stiffness of tissues can be imaged as a color image to reflect the benign and malignant tissues indirectly (13). Generally, tumor tissue has a harder texture relative to normal tissue. Research found that elastography had a better diagnostic efficiency compared with single grayscale or blood flow Doppler feature (14, 15).

PET/CT and elastography are useful tools in the diagnosis of intrathoracic LNs, which can help the selection of LNs with the greatest likelihood of malignancy during EBUS-TBNA and reduce unnecessary puncture (16, 17). For LNs with negative TBNA results, PET/CT and elastography may have a good supplementary role to reduce FNR. The two methods can reflect the benign and malignant LNs from different aspects, such as elastography, which mainly reflects the degree of stiffness of LNs, while PET/CT mainly reflects the degree of FDG metabolism of LNs. However, there has been no related research about the comparison of PET/CT and elastography, as well as the combination of the two methods in predicting intrathoracic malignant LNs. This study aimed to analyze PET/CT and EBUS elastography indicators in the model group to compare the diagnostic efficiency of the two methods for intrathoracic LNs. Then, a prediction model will be established based on the model group and the diagnostic efficiency will be validated in another dataset.

## Materials and methods

### Patients

This study was conducted in Shanghai Chest Hospital. EBUS-TBNA examination was performed on patients who met the following criteria: (1) enlarged mediastinal/hilar LNs (at least 1 node >10 mm in the short axis) based on CT or positive intrathoracic LNs detected (SUV ≥ 2.5) by PET/CT; (2) pathological confirmation by EBUS-TBNA was clinically required and feasible to confirm the nature of the LN; and (3) no contraindication to the procedure. Patients who underwent EBUS-TBNA examination and PET/CT from March 2018 to November 2019 were consecutively enrolled and LNs meeting the following criteria were analyzed: The time interval between PET/CT and EBUS-TBNA was less than 1 month; LNs had not been diagnosed before EBUS-TBNA examination and no antitumor therapy had been performed for target LNs before EBUS-TBNA or PET/CT. LNs without elastography videos were excluded. LNs from March 2018 to June 2019 were assigned into the model group and LNs from July 2019 to November 2019 were assigned into the validation group. This study was approved by the local Ethics Committee of Shanghai Chest Hospital (No. KS-1947). The final diagnosis of LNs depended on pathological results of EBUS-TBNA, thoracoscopy, mediastinoscopy, microbiological examination, or clinical follow-up for at least 1 year.

### ^18^F-FDG PET/CT image acquisition

All patients were intravenously injected with 0.10–0.15 mCi/kg (3.7–5.6 Mbq/kg) of ^18^F-FDG after fasting for 6 h with a blood glucose level of less than 10.0 mmol/L (180 mg/dl). A combined PET/CT scanner Biograph 64, Siemens, Germany was used 45–60 min later after ^18^F-FDG injection. Patients were subjected to CT positioning scanning from the skull base to one-third of the upper femur and then the scanned area was selected for spiral CT scanning. Scanning conditions were as follows: tube voltage was 120 kV, tube current was automatically adjusted according to CARE Dose 4D technology, and layer thickness was 5.0 mm. Subsequently, 5–6 beds were used for whole-body PET image acquisition, and the acquisition time was 2 min/bed. CT scan data were used to correct the attenuation of PET images, and the TrueX + TOF method was used to reconstruct the images to obtain PET images, CT images, and transverse, sagittal, and coronal fusion images (18).

### Elastography

Elastography videos of LNs were recorded with an ultrasound host (EU-ME2, Olympus) and an ultrasound bronchoscope (BF-UC260FW, Olympus) in accordance with standard operation. A scanning frequency of 10 MHz for the ultrasound probe was set for all LNs. After grayscale and Doppler mode were examined, a bronchoscopist switched to elastography mode. The sampling frame should include the target LN and surrounding tissue. When the EBUS probe touched the airway, internal compression of targeted LN from fluctuation of adjacent vessels and the breathing movement can exert a pressurization effect to form elastography. If the image is not ideal, the operator can gently press the screw part of the bronchoscopy handle to pressurize the airway at a frequency of 3–5 times per second to achieve ideal images. After the elastography became stable, two 20-s videos were recorded and stored (14).

### Measurement of PET/CT and elastography parameters

All PET/CT parameters were firstly measured by a nuclear medicine physician with more than 3,000 cases of PCT/CT imaging diagnostic experience and then reviewed by another nuclear medicine physician with a similar experience. Short axis was measured at the maximum cross-section of the targeted LN on the CT image (1). Functional images of the maximal standardized uptake value (SUVmax) and mean standardized uptake value (SUVmean) were obtained using attenuation-corrected transaxial images, the ^18^F-FDG injected dose, the patient’s body weight, and the cross-calibration factor between PET and the dose calibrator. SUV was defined as follows: SUV = tissue concentration (MBq/g)/[injected dose (MBq)/body weight (g)]. Siemens syngo *via* software was used to automatically calculate the metabolic tumor volume (MTV) and tumor lesion glycolysis (TLG) (TLG = SUVmean * MTV). The measurement of all parameters was based on the delineation of target LN (19).

The qualitative grading score method was used: 1 (scattered soft, mixed green–yellow–red); 2 (homogeneous soft, predominantly green); 3 (intermediate, mixed blue–green yellow–red); 4 (scattered hard, mixed blue–green); and 5 (homogeneous hard, predominantly blue). A score of 1–3 denotes benign and a score of 4–5 indicates malignant (14, 20). Three experienced doctors (LW, JC, and XZ) with EBUS imaging observation of more than 500 LNs reviewed elastography videos twice independently blind to the final diagnosis of LNs, and determined the final qualitative evaluation result of each expert subsequently. For grading score of disagreement, three experts reached a consensus to decide on the final assessment result. In order to reduce subjectivity and quantify tissue elasticity, the above three doctors selected the representative images of LNs from videos together according to the final grading score previously determined. Software developed by MatlabTM was used by two doctors (JC and XZ) together to draw the region of interest (ROI), and stiff area ratio (SAR), RGB, mean hue value, and mean gray value methods were used as the quantitative indicators (14, 21–23).

### Statistical analysis

Receiver operating characteristic analysis was used to determine the optimum cutoff values of continuous variables, and the best cutoff values were taken at the maximal Youden index. Chi-squared test or Fisher’s exact test was used for categorical variables. *p* < 0.05 was considered statistically significant. Significant PET/CT and elastography variables of the univariate analysis or those deemed clinically important were then entered into a multivariable logistic regression model to assess the factors independently associated with predict malignancy. Cohen’s kappa method was used to analyze the intra- and interobserver agreement of real-time elastography grading score (24). SPSS version 25.0 (IBM Corp., Armonk, NY, USA) was used for statistical analyses.

## Results

### Patients and LNs

A total of 154 LNs, namely, 92 malignant LNs and 62 benign LNs, from 135 patients were analyzed in the model group (**Table 1**). Adenocarcinoma accounted for the largest proportion of malignant LNs (27.27%), as well as nonspecific lymphadenitis of benign LNs (33.77%). There were 53 LNs from 47 patients in the validation group, namely, 36 malignant LNs (18 adenocarcinoma, 6 squamous carcinoma, 1 non-small cell lung cancer not otherwise specified, 5 small cell lung cancer, 3 neuroendocrine tumor not otherwise specified, 2 unknown type of lung cancer, and 1 lymphoma) and 17 benign LNs (12 nonspecific lymphadenitis, 3 sarcoidosis, and 2 tuberculosis). Perfect agreement was reached for intra- and interobserver agreement of the elastography grading score, which were 0.883 and 0.913, respectively.

**Table 1:** Patients and LNs in the model group.

Number of patients	135
Sex, male/female	94/41
Age, years, mean (range)	62.08 (35–83)
**Total LNs**	154
**Station**	**No. of LNs (%)**
2R	1 (0.65)
3P	1 (0.65)
4L	12 (7.79)
4R	47 (30.52)
7	51 (33.12)
10L	3 (1.95)
10R	6 (3.90)
11L	16 (10.39)
11Ri	9 (5.814)
11Rs	8 (5.19)
**Diagnosis**	
**Malignant**	92 (59.74)
Adenocarcinoma	42 (27.27)
Squamous carcinoma	18 (11.69)
Non-small cell lung cancer not otherwise specified	6 (3.90)
Small cell lung cancer	16 (10.39)
Neuroendocrine tumor not otherwise specified	3 (1.95)
Lymphoepithelioma-like carcinoma	1 (0.65)
Unknown type of lung cancer	2 (1.30)
Metastatic tumors (non-lung primary malignancy)	4 (2.60)
**Benign**	62 (40.26)
Nonspecific lymphadenitis	52 (33.77)
Sarcoidosis	7 (4.55)
Tuberculosis	2 (1.30)
Non-tuberculous mycobacterium infection	1 (0.65)

LNs, lymph nodes.

### Diagnostic value of PET/CT and elastography parameters

**Figure 1:**
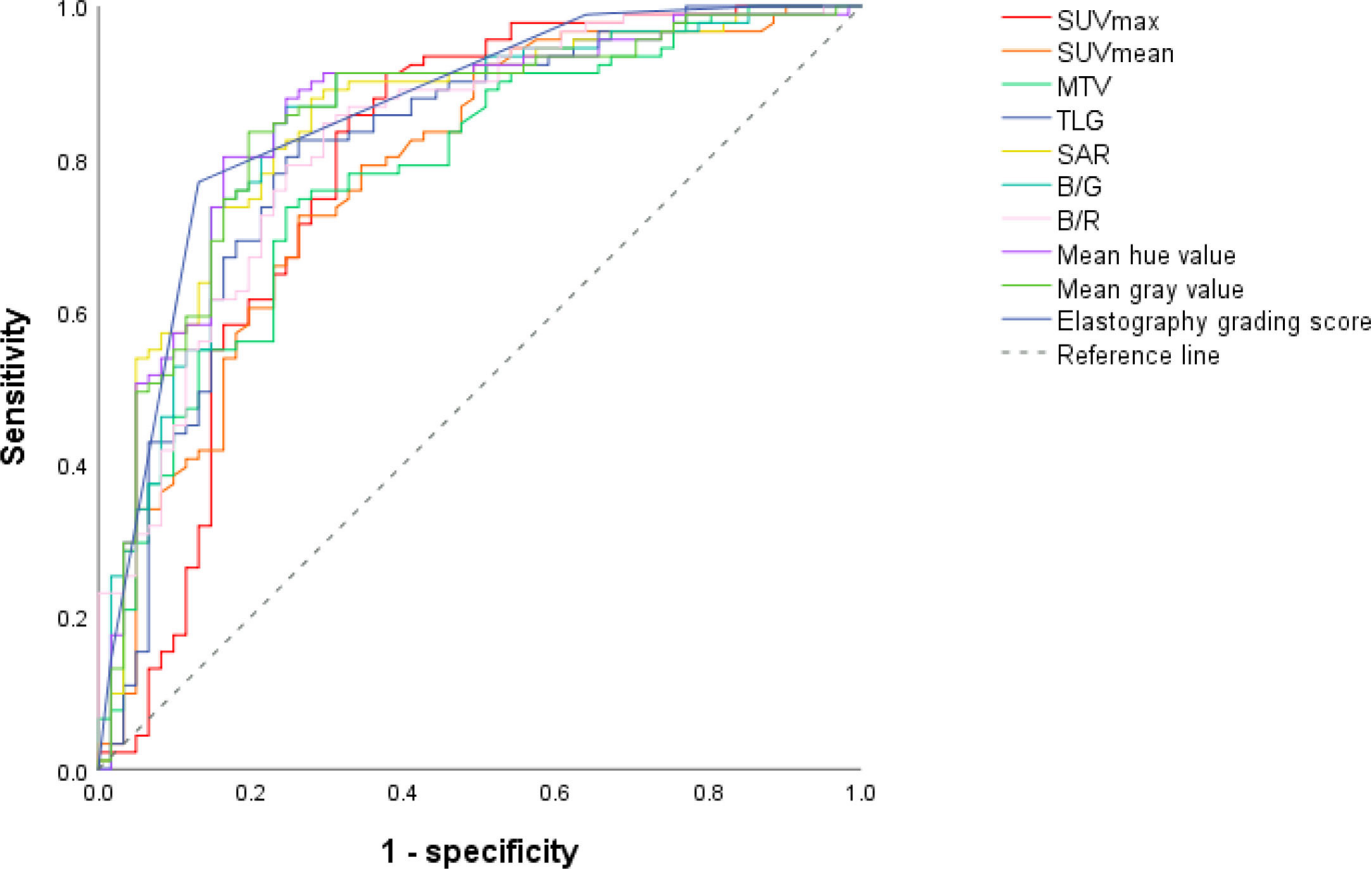
ROC curves of PET/CT and elastography parameters in the model group. Through the ROC curves, the best cutoff value reflecting the best diagnostic performance and the AUC value reflecting the overall diagnostic performance of each variable can be obtained. ROC, receiver operating characteristic; AUC, area under the curve; SUVmax, maximal standardized uptake value; SUVmean, mean standardized uptake value; MTV, metabolic tumor volume; TLG, tumor lesion glycolysis; SAR, stiff area ratio; B/G, blue versus green; B/R, blue versus red.

Receiver operating characteristic curves of PET/CT and elastography variables derived from the model group according to the final diagnosis were shown in **Figure 1**. The cutoff values and area under the curve (AUC) values of PET/CT and elastography variables were presented in **Table 2**. SUVmax and TLG were two PET/CT parameters with the highest AUC values of 0.788 and 0.813, respectively. Mean hue value and grading score were two elastography quantitative indicators with the highest AUC values of 0.854 and 0.860, respectively. As shown in **Table 3**, SUVmax, TLG, mean hue value, and grading score were independent predictive indexes for malignant LNs.

**Table 2:** Cutoff values and corresponding AUC of PET/CT and elastography variables for malignant LN prediction in the model group.

Index	Cutoff	*p*-value	AUC	95% confidence interval
SUVmax	7.02	1.77E-09	0.788	0.706–0.871
SUVmean	4.59	5.01E-09	0.780	0.703–0.857
MTV	3.47	4.68E-09	0.781	0.706–0.856
TLG	12.53	6.37E-11	0.813	0.740–0.886
SAR	0.33	4.44E-13	0.847	0.782–0.912
B/G	1.05	7.50E-13	0.844	0.777–0.910
B/R	1.47	8.84E-12	0.827	0.760–0.894
Mean hue value	132.73	1.62E-13	0.854	0.789–0.918
Mean gray value	193.39	4.44E-13	0.847	0.781–0.913
Elastography grading score	3.5	6.16E-14	0.860	0.798–0.922

AUC, area under the curve; LNs, lymph nodes; SUVmax, maximal standardized uptake value; MTV, metabolic tumor volume; SUVmean, mean standardized uptake value; TLG, tumor lesion glycolysis; SAR, stiff area ratio; B/G, blue versus green; B/R, blue versus red.

**Table 3:** Univariate and multivariate analyses of PET/CT and elastography parameters in the model group.

Index	Univariate analysis	Multivariate analysis
SUVmax > 7.02	7.82E-13	3.54E-02
SUVmean > 4.59	2.11E-08	
MTV > 3.47	2.99E-09	
TLG > 12.53	5.33E-12	3.63E-02
SAR > 0.33	1.72E-14	
B/G > 1.05	6.17E-14	
B/R > 1.47	7.74E-12	
Mean hue value > 132.73	3.85E-15	1.81E-02
Mean gray value > 193.39	7.31E-15	
Short axis > 1 cm	1.25E-03	
Elastography grading score > 3	5.40E-17	1.49E-03

SUVmax, maximal standardized uptake value; SUVmean, mean standardized uptake value; MTV, metabolic tumor volume; TLG, tumor lesion glycolysis; SAR, stiff area ratio; B/G, blue versus green; B/R, blue versus red.

### Comparison and combined diagnostic value of PET/CT and elastography

**Figure 2:**
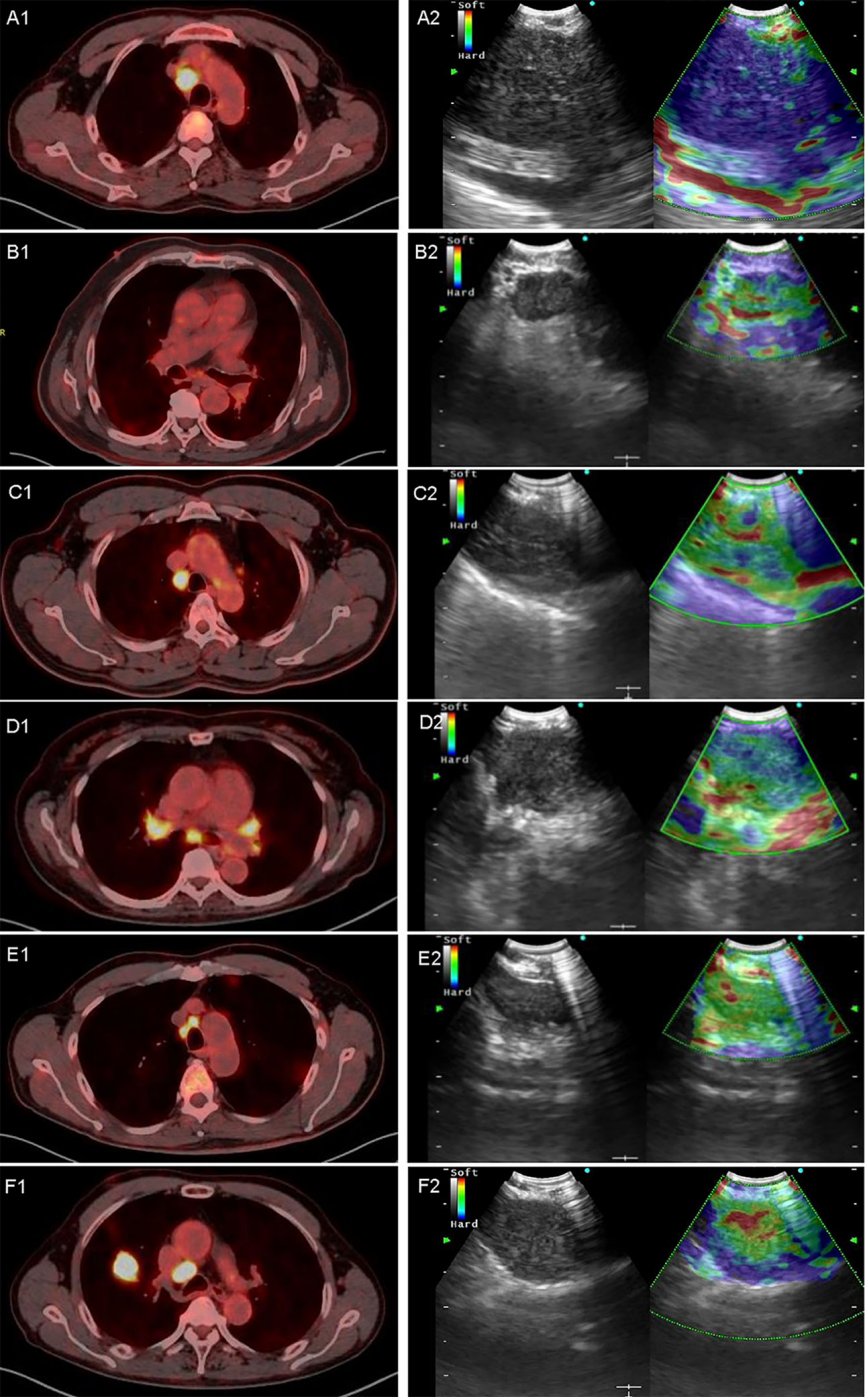
Representative images of PET/CT and elastography in a variety of diseases. A1 and A2 are representative true-positive images of malignant LNs examined by PET/CT and elastography, which showed a 4R LN with adenocarcinoma; B1 and B2 show a 7 LN with nonspecific lymphadenitis, true-negative results in both elastography and PET/CT; C1 and C2 show a 4R LN with tuberculosis, and PET/CT showed high metabolism with an SUVmax of 7.85 and a TLG of 39.09. In contrast, elastography showed a grading score of 3 and a mean hue value of 117.74; D1 and D2 showed a 7 LN with sarcoidosis, in which PET/CT showed a high metabolism while elastography showed soft tissue. SUVmax, TLG, grading score, and mean hue value were 5.5, 22.06, 3, and 105.84, respectively; E1 and E2 showed a 4R LN with nonspecific lymphadenitis, false positive by PET/CT but true negative by elastography, and SUVmax, TLG, grading score, and mean hue value were 7.84, 11.33, 1, and 89.37, respectively; F1 and F2 show a 4R LN with neuroendocrine tumor not otherwise specified, and PET/CT showed high metabolism with an SUVmax of 13.29 and a TLG of 42.19. However, elastography shows false-negative results with grading score of 3 and mean hue value of 124.42. LN, lymph node; SUVmax, maximal standardized uptake value; TLG, tumor lesion glycolysis.

**Figure 3:**
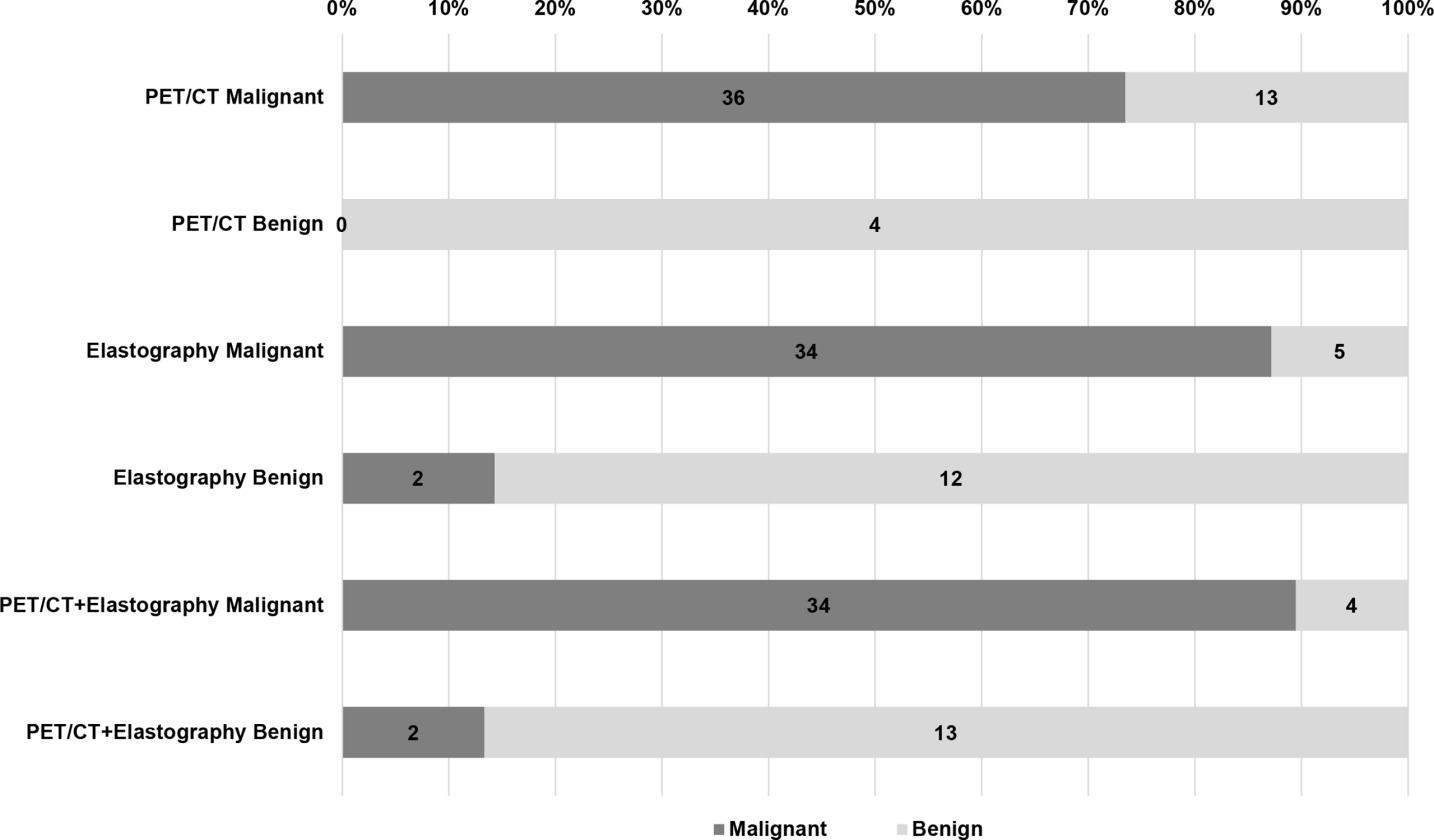
Diagnostic efficiency of PET/CT, elastography, and combination method in the validation group. Of the 53 LNs in the validation group, there were 13 false positive and 0 false negative LNs in PET/CT, 5 false positive and 2 false negative LNs in elastography, but only 4 false positive and 2 false negative LNs in the combined method. LN, lymph node.

In the model group, for PET/CT parameters, SUVmax and TLG had the highest sensitivity of 91.30% and 81.52% and the lowest FNR of 8.70% and 18.48%, respectively (**Table 4**). When SUVmax or TLG positive (SUVmax > 7.02 or TLG > 12.53) was diagnosed PET/CT positive, both SUVmax and TLG negative (SUVmax ≤ 7.02 and TLG ≤ 12.53) were diagnosed as PET/CT negative, and the sensitivity, FNR, and diagnostic accuracy of PET/CT were 93.48%, 6.52%, and 79.22%, respectively (**Table 5**). For elastography indicators, mean hue value and grading score had the highest specificity of 83.87% and 87.10%, and the lowest FPR of 16.13% and 12.90%, respectively (**Table 4**). When mean hue value or grading score positive (mean hue value > 132.73 or grading score > 3) was diagnosed as elastography positive, that is, both mean hue value and grading score negative (mean hue value ≤ 132.73 or grading score ≤ 3) were justified as elastography negative, the diagnostic accuracy of elastography was 85.71% (**Table 5**). **Figure 2** displays the false-positive representative images of PET/CT in LNs with tuberculosis, sarcoidosis, and nonspecific lymphadenitis, as well as the false-negative representative image of elastography in LNs with neuroendocrine tumor not otherwise specified. The combination of PET/CT with elastography can achieve the highest diagnostic accuracy of 87.01% in the model group; only when both the two methods (PET/CT and elastography) were positive was the combined method justified as positive. That is, either PET/CT or elastography negative was the combined method justified as negative. Similar results can be seen at the validation group, PET/CT combined with elastography had the best diagnostic performance with an accuracy of 88.68% (**Table 5**). The detailed diagnostic results of PET/CT, elastography, and the combined method in the validation group were displayed in **Figure 3**.

**Table 4:** Diagnostic efficiency of PET/CT and elastography variables for malignant lymph nodes prediction in the model group.

Index	Sensitivity		Specificity	PPV	NPV	Accuracy	FPR	FNR
SUVmax	91.30%	62.90%		78.50%	82.98%	79.87%	37.10%	8.70%
SUVmean	71.74%	74.19%		80.49%	63.89%	72.73%	25.81%	28.26%
MTV	72.83%	75.81%		81.71%	65.28%	74.03%	24.19%	27.17%
TLG	81.52%	74.19%		82.42%	73.02%	78.57%	25.81%	18.48%
SAR	88.04%	72.58%		82.65%	80.36%	81.82%	27.42%	11.96%
B/G	86.96%	72.58%		82.47%	78.95%	81.17%	27.42%	13.04%
B/R	83.70%	70.97%		81.05%	74.58%	78.57%	29.03%	16.30%
Mean hue value	80.43%	83.87%		88.10%	74.29%	81.82%	16.13%	19.57%
Mean gray value	82.61%	80.65%		86.36%	75.76%	81.82%	19.35%	17.39%
Short axis > 1 cm	95.65%	20.97%		64.23%	76.47%	65.58%	79.03%	4.35%
Elastography grading score	81.52%	87.10%		90.36%	76.06%	83.77%	12.90%	18.48%

LNs, lymph nodes; SUVmax, maximal standardized uptake value; SUVmean, mean standardized uptake value; MTV, metabolic tumor volume; TLG, tumor lesion glycolysis; SAR, stiff area ratio; B/G, blue versus green; B/R, blue versus red; PPV, positive predictive value; NPV, negative predictive value; FPR, false-positive rate; FNR, false-negative rate.

**Table 5:** The diagnostic efficiency of PET/CT, elastography, and combination model in the model and validation groups.

Index	Sensitivity	Specificity	PPV	NPV	Accuracy	FPR	FNR
Model group							
PET/CT	93.48%	58.06%	76.79%	85.71%	79.22%	41.94%	6.52%
Elastography	89.13%	80.65%	87.23%	83.33%	85.71%	19.35%	10.87%
PET/CT+ Elastography	85.87%	88.71%	91.86%	80.88%	87.01%	11.29%	14.13%
Validation group							
PET/CT	100.00%	23.53%	73.47%	100.00%	75.47%	76.47%	0.00%
Elastography	94.44%	70.59%	87.18%	85.71%	86.79%	29.41%	5.56%
PET/CT+ Elastography	94.44%	76.47%	89.47%	86.67%	88.68%	23.53%	5.56%

PPV, positive predictive value; NPV, negative predictive value; FPR, false-positive rate; FNR, false-negative rate.

## Discussion

This study explored the diagnostic capacity of PET/CT and EBUS elastography on intrathoracic LNs. SUVmax ≥ 2.5 was commonly used as a positive criterion, and sensitivity and specificity estimates for the SUVmax ≥ 2.5 were 81.3% and 79.4%, respectively (25). The sensitivity of SUVmax was 91.30% in this study. However, the high SUVmax values of sarcoidosis and tuberculosis, which were 13.53 ± 8.37 and 17.57 ± 10.89, respectively, led to a low specificity of 62.90%. FDG uptake is related to the size of LNs, and a false-negative result could be caused by the small size of LNs (26). In the model group, SUVmax for short axis ≤ 1 cm and >1 cm is 6.01 ± 3.53 and 11.00 ± 5.36, respectively, with significant statistical difference (*p* < 0.001). It was suggested that SUVmax is more reproducible than SUVmean (27). In our study, SUVmean had the lowest accuracy among PET/CT parameters. Volumetric parameter MTV could be used to predict LN metastasis in lung cancer, and it is an important prognostic indicator for NSCLC (28). MTV in our study showed statistically significant differences in benign and malignant LNs, but had the lowest AUC value among the four PET/CT parameters. TLG is calculated by multiplying SUVmean to MTV in an ROI, which can represent both metabolic and volumetric information (19). TLG is an independent parameter for differentiating benign and malignant LNs with the highest AUC among PET/CT parameters in this study. In both model and validation groups, the combination of SUVmax and TLG provided better diagnostic efficiency than any single parameter.

CP-EBUS sonographic features can be used to predict malignant and benign diagnoses during EBUS-TBNA (12). By measuring the compressibility of the tissue, elastography can reflect the different relative stiffness between normal and malignant LNs, and it has been extensively studied using qualitative and quantitative methods. The elastography grading score divides an image into 5 grades, which is convenient for clinical application. For quantitative elastography indicators, SAR is a good predictor of malignant LNs, with an accuracy of 81.82% in this study and 83% and 82.35% in other studies (22, 29). The RGB color model defines a color density of 0–49 as blue pixels. In the model group, blue versus green (B/G) and blue versus red (B/R) had accuracies of 81.17% and 78.57%, respectively, but with the lowest AUC among quantitative elastography indicators. The HSV color model defines a pixel value range from 145 to 180 as blue pixels. Mao et al. found that the AUC of mean hue value was 0.814, and when the cutoff value was 126.28, the corresponding accuracy was 80.88% (22). Mean hue values were 145.00 ± 16.16 and 119.66 ± 17.74 for malignant and benign LNs, and it was the only independent predictor among quantitative indicators. Mean gray values for malignant and benign LNs in the model group were 197.36 ± 14.06 and 182.55 ± 29.61. The AUC of mean gray value was lower than that of mean hue value in this study. In terms of diagnostic methods, the combination of qualitative score with mean hue value had a better diagnostic performance in both groups relative to PET/CT.

^18^F-FDG is not specific for tumor, which can be taken up by various physiologic variants and benign pathologic lesions, leading to false-positive results (30, 31). In the model group, the FPR of PET/CT (SUVmax combined with TLG) was 41.94% (26/62), and 73.08% (19/26) were diagnosed as true negative by elastography among the 26 false-positive cases by PET/CT (19 nonspecific lymphadenitis, 4 sarcoidosis, 2 tuberculosis, and 1 non-tuberculous mycobacterium infection). Inflammation was a well-known factor associated with FPR of PET scan. For nonspecific lymphadenitis, 36.54% (19/52) in the model group and 66.67% (8/12) in the validation group were diagnosed as false positive by PET/CT, and the proportions for sarcoidosis were 57.14% (4/7) in the model group and 100% (3/3) in the validation group. For tuberculosis, a total of 4 cases in model and validation groups had a positive SUVmax. Studies had shown that tuberculosis is prone to lead to false-positive results, because along with the accumulation of FDG in inflammatory phagocytes and macrophages, glucose metabolism increased, and staging accuracy using PET/CT was low in lung cancer patients with parenchymal tuberculosis sequelae (6, 32). In the validation group, there were 13 cases of false-positive diagnosis by PET/CT, among which 9 cases were diagnosed as negative by elastography. This result suggested that elastography could reduce the FPR of PET/CT. Besides, 2 cases of false negative results diagnosed by elastography (1 neuroendocrine tumor not otherwise specified and 1 adenocarcinoma) showed high FDG uptake of SUVmax, suggesting that PET/CT may reduce FNR of elastography. However, only when PET/CT and elastography all positive in this study were considered as malignant, so the combined method did not decrease the FNR.

This study still had some limitations. Although the diagnostic model constructed in this study has been validated, the validation part was still a retrospective study, and further prospective validation is needed. Moreover, all LNs in this study were from a specialized thoracic hospital with a limited category of diseases and the dataset was not large enough. Therefore, a multicenter study with a larger dataset may acquire better results because of different case compositions in different research centers. Moreover, the subjects of this study were mainly patients undergoing LN diagnosis rather than lung cancer staging, such as sarcoidosis and tuberculosis in benign diseases, which may be the reason why the diagnostic specificity and accuracy of PET/CT were slightly lower than those of elastography.

In conclusion, the non-invasive diagnostic model combining PET/CT and EBUS elastography constructed in this study had a higher diagnostic accuracy than any single method for intrathoracic benign and malignant LN differentiation. Furthermore, the diagnostic performance of elastography was superior to PET/CT when the two methods were compared separately. This study may optimize the clinical diagnostic methods of intrathoracic benign and malignant LNs.

## Data availability statement

The original contributions presented in the study are included in the article/supplementary material. Further inquiries can be directed to the corresponding authors.

## Ethics statement

This study was reviewed and approved by Ethics Committee of Shanghai Chest Hospital. Written informed consent for participation was not required for this study in accordance with the national legislation and the institutional requirements.

## Author contributions

XZ processed elastography videos, analyzed elastography sonographic features collected statistics, and drafted and revised the manuscript. XS collected and analyzed PET/CT data and drafted the manuscript. JC performed EBUS-TBNA examination, analyzed elastography indicators. LW analyzed the elastography indicators. LY assisted in the final diagnostic of lymph nodes. YL revised the paper. WX designed the conception of the study and revised the work. JS designed the conception of the study, revised the manuscript, and supported this study. All authors contributed to the article and approved the submitted version.

## Funding

This work was supported by the National Natural Science Foundation of China [grant number 81870078], the Shanghai Municipal Health and Medical Talents Training Program [grant number 2018BR09], and the Shanghai Municipal Education Commission-Gaofeng Clinical Medicine Grant Support [grant number 20181815].

## Acknowledgments

The authors acknowledge the doctors in the Department of Respiratory and Critical Care Medicine for the collection of elastography videos and the doctors in the Nuclear Department for the acquisition of PET/CT imaging at Shanghai Chest Hospital.

## Conflict of interest

All authors in this study declare that the research was conducted in the absence of any commercial or financial relationships that could be construed as a potential conflict of interest.

## Publisher’s note

All claims expressed in this article are solely those of the authors and do not necessarily represent those of their affiliated organizations, or those of the publisher, the editors and the reviewers. Any product that may be evaluated in this article, or claim that may be made by its manufacturer, is not guaranteed or endorsed by the publisher.
